# Getting there: understanding the chromosomal recruitment of the AAA+ ATPase Pch2/TRIP13 during meiosis

**DOI:** 10.1007/s00294-021-01166-3

**Published:** 2021-03-12

**Authors:** Richard Cardoso da Silva, Gerben Vader

**Affiliations:** 1 Department of Mechanistic Cell Biology, Max Planck Institute of Molecular Physiology, Otto-Hahn-Strasse 11, 44227 Dortmund, Germany; 2 Present Address: Department of Molecular Mechanisms of Disease, University of Zürich, Winterthurerstrasse 190, 8057 Zürich, Switzerland; 3 Present Address: Department of Clinical Genetics, Section of Oncogenetics, Cancer Center Amsterdam, De Boelelaan 1118, 1081 HV Amsterdam, The Netherlands

**Keywords:** Meiosis, Chromosome synapsis, Pch2, TRIP13, Recombination, Checkpoint, rDNA

## Abstract

The generally conserved AAA+ ATPase Pch2/TRIP13 is involved in diverse aspects of meiosis, such as prophase checkpoint function, DNA break regulation, and meiotic recombination. The controlled recruitment of Pch2 to meiotic chromosomes allows it to use its ATPase activity to influence HORMA protein-dependent signaling. Because of the connection between Pch2 chromosomal recruitment and its functional roles in meiosis, it is important to reveal the molecular details that govern Pch2 localization. Here, we review the current understanding of the different factors that control the recruitment of Pch2 to meiotic chromosomes, with a focus on research performed in budding yeast. During meiosis in this organism, Pch2 is enriched within the nucleolus, where it likely associates with the specialized chromatin of the ribosomal (r)DNA. Pch2 is also found on non-rDNA euchromatin, where its recruitment is contingent on Zip1, a component of the synaptonemal complex (SC) that assembles between homologous chromosomes. We discuss recent findings connecting the recruitment of Pch2 with its association with the Origin Recognition Complex (ORC) and reliance on RNA Polymerase II-dependent transcription. In total, we provide a comprehensive overview of the pathways that control the chromosomal association of an important meiotic regulator.

## Meiosis

Sexual reproduction requires the successful production of viable gametes. The production of these highly specialized cells involves a dedicated cell division process (i.e., meiosis), during which several unique processes are executed. Many key events take place during a protracted G2/prophase of the meiotic program when homologous recombination (HR) between homologous chromosomes occurs (Subramanian and Hochwagen 2014). Recombination is initiated by the programmed formation of double-strand breaks (DSBs), catalyzed by the topoisomerase-like protein Spo11 (Keeney et al. 2014). Repair of DSBs to form crossovers (COs) is needed to form physical links between homologous chromosomes which enables successful meiotic chromosome segregation (Humphryes and Hochwagen 2014). During meiotic G2/prophase, individual homologous chromosomes are arranged into a meiosis-specific organization consisting of an array of chromatin loops that emanate from a proteinaceous chromosome axis (Zickler and Kleckner 1999). In budding yeast, this chromosomal axis is established on cohesin complexes (containing the meiosis-specific kleisin subunit Rec8) (Klein et al. 1999). In addition to cohesin, it contains the HORMA-domain protein Hop1 and the coiled coil-containing protein Red1 (de los Santos and Hollingsworth 1999; Hollingsworth et al. 1990; Hollingsworth and Ponte 1997; Klein et al. 1999; Smith and Roeder 1997; Sym et al. 1993). An additional axis-component is Mek1, a meiosis-specific protein kinase that functions in the meiotic checkpoint and promotes repair of DSBs using sequences present on homologous chromosomes (Hollingsworth and Ponte 1997; Niu et al. 2005; Wan et al. 2004). This specialized chromosome architecture facilitates DNA break activity, checkpoint function, and interhomolog-based recombinational CO repair in meiotic G2/prophase (Carballo et al. 2008; Humphryes and Hochwagen 2014; Panizza et al. 2011; Zickler and Kleckner 1999). Another process that typifies meiotic G2/prophase is the engagement and eventual synapsis of homologous chromosomes. Synapsis is defined by the controlled appearance of a structurally conserved proteinaceous zipper-like structure, named the synaptonemal complex (SC), in between paired homologs.

Throughout meiotic G2/prophase, DSB activity, CO establishment, and chromosome synapsis need to be coordinated, and several regulatory factors ensure that these events are faithfully executed. In this review, we focus on one such factor: Pch2/TRIP13, and discuss how the chromosomal recruitment of this factor is regulated, and how recruitment of Pch2/TRIP13 is linked to meiosis-specific chromosomal events.

### The role and biochemistry of Pch2/TRIP13

Pch2 (for Pachytene CHeckpoint 2, alternatively called TRIP13 in mammals; we thus occasionally refer to this enzyme as Pch2/TRIP13) is a member of the AAA+ ATPase (AAA+ ATPases associated with diverse cellular activities) enzyme family (Hanson and Whiteheart 2005). This enzyme is widely conserved and affects a multitude of events during meiosis, such as cell cycle checkpoint function, local DSB activity, CO formation, and chromosome morphogenesis (Bhalla and Dernburg 2005; Borner et al. 2008; Herruzo et al. 2016; Joshi et al. 2009, 2015,2019; Joyce and McKim 2009,2010; Li and Schimenti 2007; Raina and Vader 2020; San-Segundo and Roeder 1999; Subramanian et al. 2016; Vader et al. 2011; Wojtasz et al. 2009; Wu and Burgess 2006; Zanders and Alani 2009). Thus, Pch2/Trip13 is an important regulator of meiotic processes, and mutation of the TRIP13 gene causes infertility in humans (Zhang et al. 2020).

AAA + ATPases leverage the energy derived from ATP hydrolysis to mechanically remodel client molecules (Hanson and Whiteheart 2005; Puchades et al. 2020). Biochemical and structural analysis has revealed that Pch2 acts on client proteins that contain an HORMA domain (Alfieri et al. 2018; Chen et al. 2014; Ye et al. 2017). The HORMA domain [for Hop1, Rev7, and Mad2 (Aravind and Koonin 1998)] is a structural domain that can adopt distinct topological states (Mapelli et al. 2007; Rosenberg and Corbett 2015). In what is considered the active state of HORMA-domain-containing proteins, the COOH-terminal region of the HORMA domain embraces a short peptide motif [termed closure motif (CM)] present in proteins (Rosenberg and Corbett 2015). This binding, referred to as a ‘safety belt’-type binding, generates a topological interaction between a ‘closed’ HORMA domain and a CM-containing factor. This ‘closed’ HORMA topology can be reversed into an ‘open/unbuckled’ topology, a transition that generally will lead to the release of the CM-containing peptide and thus disruption of the HORMA/CM-dependent complex assembly. The transition of ‘closed’ to ‘open’ HORMA topology requires energy which is provided by Pch2/TRIP13 AAA + activity. Pch2/TRIP13 activity promotes dissolution of HORMA/CM-based complex formation, and as such, its enzymatic activity can have profound effects on signaling cascades that rely on HORMA-based complex assembly (Musacchio 2015; Rosenberg and Corbett 2015). Substrates for Pch2/TRIP13 in meiotic prophase are meiotic HORMA-domain proteins (known as Hop1 in budding yeast, ASY1 in *Arabidopsis thaliana,* HIM-3, and HTP-1/2/3 in *Caenorhabditis elegans*, and HORMAD1/2 in mammals). HORMADs are components of the meiotic chromosome axis (see above), and incorporation of Hop1 into a biochemical complex with Red1 and Mek1 (here referred to as RHMc) depends on CM-mediated interaction between Red1 and Hop1 (Friedman et al. 1994; Niu et al. 2005; West et al. 2019, 2018). Regulated association/dissociation of Hop1 with chromosomes is crucial for DSB activity, crossover recombination, checkpoint control, and chromosome organization (Rosenberg and Corbett 2015). Recruitment of Pch2 to chromosomes is associated with removal of Hop1 from chromosomes and with diminished Hop1/RHMc-related activity (Borner et al. 2008; Raina and Vader 2020; Subramanian et al. 2016, 2019). Based on these observations and the known biochemical activity of Pch2/TRIP13 toward HORMA domains (Alfieri et al. 2018; Chen et al. 2014; Ye et al. 2017), a model emerges in which a main role of Pch2, when recruited to chromosomes, is to dismantle RHMc via its enzymatic activity toward this HORMA-domain-containing complex.

Biochemical and cell biological analyses have shown that a protein called p31comet acts as an adaptor of Pch2/TRIP13. p31comet is an HORMA-domain-containing protein (Yang et al. 2007) and is key in enabling the interaction of Pch2/TRIP13 with other HORMA proteins, such as Mad2 and Rev7 (Clairmont et al. 2020; Eytan et al. 2014; Ma and Poon 2016; Miniowitz-Shemtov et al. 2015; Sarangi et al. 2020; Ye et al. 2015, 2017). Recent work demonstrated that in plants (*A. thaliana* and *Oryza sativa*), as well as *C. elegans,* p31comet is also involved in Pch2/TRIP13 mediated effects on meiotic HORMADs (Balboni et al. 2020; Giacopazzi et al. 2020; Ji et al. 2016). In *A. thaliana*, p31comet is needed for the recruitment of Pch2/TRIP13 to chromosomes (Balboni et al. 2020), but in *C. elegans*, this does not seem to be the case (Balboni et al. 2020; Giacopazzi et al. 2020). No clear p31comet homolog has been described in budding yeast. In this organism, an adaptor role of p31comet might be executed via alternative pathways. In line with such reasoning, budding yeast Pch2 has been described to functionally interact with defined chromosome-associated factors, such as Xrs2 and Orc1 (Ho and Burgess 2011; Vader et al. 2011; Villar-Fernandez et al. 2020), suggesting that these factors might perform adaptor-like functions (see more below).

To appreciate and understand how Pch2 function controls meiotic G2/prophase, it is paramount to reveal how the chromosomal association of Pch2 is controlled. Here, we summarize the current understanding of the mechanisms that influence the recruitment of Pch2 to chromosomes and discuss how chromosomal and chromatin context might affect Pch2 function during meiosis. Most of the knowledge on the chromosomal recruitment and function of Pch2 is derived from work in budding yeast meiosis. We will thus focus on work done in this model organism and will highlight insights gleaned from work in other model organisms. For more in-depth information on the functional and biochemical functions of Pch2/TRIP13 and HORMA-domain-based signaling, we refer the reader to earlier reviews (Rosenberg and Corbett 2015; Vader 2015).

### Chromosomal localization and function of Pch2

**Fig. 1:**
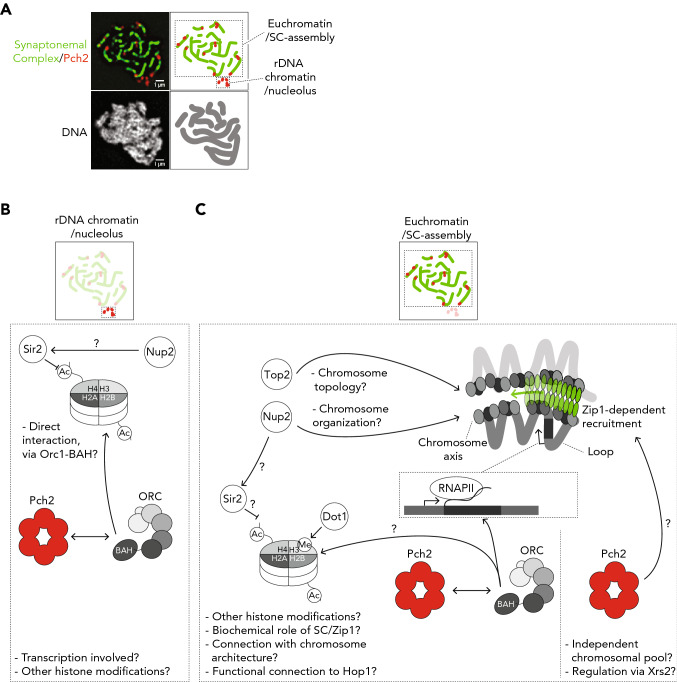
**a** Immunofluorescence microscopy image of spread chromosomes of a meiotic G2/prophase budding yeast cell, stained for Pch2 (red), the Synaptonemal Complex (Gmc2 staining; green), and DNA (gray). A representative schematic of the chromosome spread is shown, see (Cardoso da Silva et al. 2020) for experimental details. Highlighted is the specific localization of Pch2 to the nucleolar/rDNA region, and to synapsed chromosomes. **b** Model depicting the factors, centered around nucleosomes, that are known to influence Pch2 localization at the nucleolar/rDNA region. **c** Model depicting the factors and processes (RNAPII-dependent transcription, Top2, Nup2, chromatin modifications, and SC assembly) known to influence Pch2 localization to synapsed chromosome regions. Potential additional pool of Pch2 interacting with Xrs2 is also indicated. See details in the text. Pertinent questions are indicated, as discussed throughout the manuscript

The study that discovered Pch2 through a genetic screen in budding yeast (San-Segundo and Roeder 1999) first revealed its localization pattern during meiotic G2/prophase. Most prominently, Pch2 is enriched in the nucleolus, the nuclear region where the repetitive ribosomal (r)DNA is localized (Fig. 1a). In addition to this regional enrichment, Pch2 is also present as individual chromosomal foci that co-localize with synapsed chromosomes (i.e., chromosomal regions that contain polymerized SC) (Fig. 1a). Subsequent work revealed that the SC component Zip1 is required for Pch2 localization and that removal of Hop1 from chromosomes depends on Zip1 (Borner et al. 2008; Subramanian et al. 2016). Zip1, which can self-organize into higher order structures (Sym et al. 1993), is a key component of the central element of the SC (also referred to as the transverse filament), whose polymerization is a crucial step in the SC establishment. The dependence for recruitment of Pch2 to chromatin and HORMAD removal on the proper assembly on Zip1 (or its homologs) appears conserved, at least in mouse and *C. elegans* (Deshong et al. 2014; Wojtasz et al. 2009). These observations led to the model that Zip1 is a key determinant of Pch2 recruitment. The majority of (Zip1-dependent) SC polymerization initiates at a subset of DSBs which are designated to form crossovers (Storlazzi et al. 1996). These sites are defined by the association of a subset of meiotic factors collectively referred to as Synapsis Initiation Complexes (SICs) (Agarwal and Roeder 2000; Chua and Roeder 1998; Fung et al. 2004; Zickler et al. 1992). Zip1 is also a part of SICs, and as such is important in promoting crossover formation (Borner et al. 2004). The polymerization of the SC from SICs (and thus crossover-designated DSBs) is proposed to function as a chromosome-autonomous communication device that controls chromosome-associated processes such as DSB activity, CO repair, and checkpoint function. SC polymerization correlates with an attenuation of DSB activity, interhomolog-biased repair, and checkpoint function (Mu et al. 2020; Raina and Vader 2020; Subramanian et al. 2016). These events are correlated with the local removal of Hop1 (or its HORMAD homologs), and the recruitment of Pch2 (Borner et al. 2008; Wojtasz et al. 2009). The fact that Zip1 plays multiple roles during meiotic prophase (i.e., at SICs and within the polymerizing SC) makes it currently impossible to discern which of these functions is responsible for its role in recruiting Pch2. Other components of the central element of the SC, such as Ecm11 and Gmc2 (Humphryes et al. 2013), do not appear to functionally act at SIC sites (Voelkel-Meiman et al. 2016,2019). Recent work has shown that deleting these factors leads to increased DSB activity and increased CO formation (Mu et al. 2020; Voelkel-Meiman et al. 2016), which are effects that are connected to Hop1 function on chromosomes. As such, and also in light of the proposed connection between dynamic SC polymerization and Hop1 removal, the most parsimonious explanation is that the role of Zip1 in promoting Pch2 recruitment is related to its role in SC establishment. Investigating whether other SC factors influence Pch2 recruitment will provide more insights into the connection between Pch2 and SC function.

Pch2 localization to chromatin during meiotic G2/prophase thus appears regulated both regionally (i.e., via specific—continuous—recruitment to the rDNA array/nucleolus), as well as temporally (i.e., via a dependency on the successful appearance of Zip1 on non-rDNA euchromatin). Importantly, in both cases, recruitment of Pch2 is linked to Pch2-dependent removal of Hop1 from the chromosome axis (Borner et al. 2008; Raina and Vader 2020; San-Segundo and Roeder 1999; Subramanian et al. 2016, 2019), which appears to be one of the major roles of Pch2 when on chromosomes. These findings underscore that—once recruited to chromosomes—the downstream effect of Pch2 recruitment is founded on a universal biochemical logic.

The rDNA array of budding yeast is localized on chromosome XII and is composed of tandem repeats of hundreds of copies of the *35S* and *5S* rRNA. Its chromatin is highly specialized, to allow RNA Polymerase (RNAP) I- and III-dependent transcription but to minimize RNAPII-driven transcription (Moazed 2001; Nomura 2001). During meiosis, the rDNA array behaves atypically: Spo11-dependent DSB formation and, as a consequence, meiotic recombination is extremely low within the array (Gottlieb and Esposito 1989; Mieczkowski et al. 2007; Pan et al. 2011; Petes and Botstein 1977; San-Segundo and Roeder 1999; Vader et al. 2011). In addition, the rDNA does not synapse, and thus remains devoid of Zip1/SC (Dresser and Giroux 1988; Sym et al. 1993). Since elsewhere in the genome Zip1 is a key determinant of Pch2 recruitment, this observation led to the idea that the nucleolar pool of Pch2 is regulated in a manner that is molecularly distinct from the euchromatic SC-dependent pool. Indeed, some aspects of rDNA-recruitment of Pch2 seem unique, but recent work has also hinted at shared molecular characteristics driving Pch2 recruitment to the rDNA and to euchromatin. The most prominent rDNA factor that influences the local Pch2 recruitment is the histone deacetylase Sir2, which confers a specialized repressive heterochromatin-like state to the yeast rDNA (Cavero et al. 2016; Gottlieb and Esposito 1989; Mieczkowski et al. 2007; Moazed 2001; San-Segundo and Roeder 1999; Vader et al. 2011). This observation suggests a connection between the specialized (Sir2-dependent) chromatin of the rDNA and the localization of Pch2. Further strengthening the possible functional relationship between chromatin modifications and Pch2 chromosomal recruitment is the fact that Dot1, a histone methyltransferase that catalyzes the mono-, di-, and trimethylation of Histone H3K79 (van Leeuwen et al. 2002), was identified in the same genetic screen that identified Pch2 as a checkpoint factor (San-Segundo and Roeder 1999, 2000). Dot1 (and the methylation of H3K79) influences the localization pattern of Pch2 along meiotic chromosomes (Ontoso et al. 2013; San-Segundo and Roeder 1999, 2000).

The role of Pch2 within the rDNA is to suppress meiotic recombination between repetitive sequences, ostensibly to minimize genome destabilization triggered by non-allelic HR (NAHR) (San-Segundo and Roeder 1999; Vader et al. 2011). Pch2 achieves this via local suppression of DSBs within the outer regions of the rDNA, likely via a local effect on Hop1 recruitment (San-Segundo and Roeder 1999; Vader et al. 2011). To perform this function, Pch2 collaborates and directly interacts with Orc1, a subunit of the Origin Recognition Complex (ORC) (Vader et al. 2011; Villar-Fernandez et al. 2020) (Fig. 1b). Orc1/ORC directly associates with DNA and nucleosomes, in a manner that can be influenced by specific histone modifications (De Ioannes et al. 2019; Eaton et al. 2010; Gartenberg and Smith 2016; Muller et al. 2010). These findings, together with the discussed connection between Pch2 localization/function and Sir2 and Dot1 activity, hint at a connection between Pch2 and specific chromatin-associated events (Fig. 1c). We discuss these connections and their implications in more detail below. We note that enrichment of Pch2 to rDNA has not been reported outside of budding yeast, suggesting that this localization pattern might constitute a unique feature of budding yeast meiosis. However, in *Drosophila melanogaster*, Sir2 is linked to Pch2 function in meiosis (Joyce and McKim 2010), and it will be interesting to carefully examine the possible association of Pch2 with nucleolar regions in species other than budding yeast. In relation to this, a recent study in *C. elegans* revealed a role for Dot1 in checkpoint control (Lascarez-Lagunas et al. 2020), as in budding yeast (San-Segundo and Roeder 1999,2000). However, there is currently no evidence that this function is directly connected to Pch2 localization to chromosomes (or the rDNA/nucleolus) (Lascarez-Lagunas et al. 2020).

A key characteristic of the recruitment of Pch2 to (non-rDNA) regions of meiotic chromosomes is that it is correlated with chromosome synapsis, and dependent on Zip1 (Borner et al. 2004; Deshong et al. 2014; Lascarez-Lagunas et al. 2020; San-Segundo and Roeder 1999) (Fig. 1a and see above). The molecular mechanisms of Pch2 recruitment to synapsed chromosomes and its underlying role are not understood at a mechanistic level. A possibility is that Pch2 interacts with SC components such as Zip1 (Borner et al. 2008; San-Segundo and Roeder 1999; Subramanian et al. 2016). However, certain lines of evidence argue against the notion that Pch2 is recruited to chromosomes through an interaction with SC components and/or following SC assembly. First, a direct interaction between Pch2 and Zip1 has not been described. Second, a non-null allele of Zip1 (*zip1-4LA*), which retains its ability to build SCs with wild-type kinetics, impairs chromosomal recruitment of Pch2 (Mitra and Roeder 2007; Subramanian et al. 2016). Conceivably, the region that is mutated in *zip1-4LA* could constitute the interaction domain between Pch2 and Zip1*,* a notion that could in principle be testable using biochemical reconstitution. However, an alternative interpretation is that SC establishment is not the sole factor that determines Pch2 recruitment to non-rDNA chromatin—in other words, Zip1/SC is not a direct recruiter, but rather plays an ‘indirect’ regulatory role. This idea is strengthened by the observation that in the absence of Dot1, Pch2 is localized to chromosomes even in cells that lack Zip1 (i.e., in *dot1Δ zip1Δ* cells) (Ontoso et al. 2013; San-Segundo and Roeder 2000). These findings suggest that, rather, Zip1 functions as a ‘licensing’ factor that allows Pch2 recruitment, but is not strictly required, especially under certain conditions. Dynamic regulation of Pch2 recruitment might involve SC-dependent deposition of certain post-translational modifications that influence Pch2 recruitment. For example, SUMOylation is abundant in meiosis (Bhagwat et al. 2021), and SC polymerization is associated with chromosomal SUMOylation (Cheng et al. 2006; Hooker and Roeder 2006; Voelkel-Meiman et al. 2013). It is thus a possibility that SUMO-dependent modifications of currently (chromatin) factors might be involved in controlling Pch2 recruitment.

### Novel insights into recruitment of Pch2 to meiotic chromosomes

If Zip1 is not the sole factor directing chromosomal recruitment of Pch2, what is then determining Pch2 localization? As mentioned, Orc1 contributes to Pch2 recruitment and function within the rDNA/nucleolus (Herruzo et al. 2019; Vader et al. 2011). Orc1 is a subunit of the Origin Recognition Complex (ORC), a six subunit (Orc1-6) hexameric AAA + ATPase (Bell and Labib 2016). ORC forms a complex with Pch2 in vivo, and Pch2 directly binds to ORC in vitro, thus forming a meiosis-specific AAA + assembly complex (Vader et al. 2011; Villar-Fernandez et al. 2020). ORC has a broad role in chromosome replication where it binds to defined sites throughout the genome, and could thus ostensibly play a general role in Pch2 recruitment, also outside of the rDNA. In line with this idea, we recently found that inactivation of Orc1 (using *orc1-161*, a temperature-sensitive *ORC1* allele) leads to a reduction in Pch2 recruitment to euchromatin, as judged by immunofluorescence (Cardoso da Silva et al. 2020). We note that in another report, a degron-based conditional Orc1 depletion did not affect the non-nucleolar pool of Pch2 to chromosomes (Herruzo et al. 2019). In this study, the authors also analyzed the association of Pch2 with extrachromosomal SC aggregates, called polycomplexes (PCs). They found that Orc1 was not required for the association of Pch2 with (Zip1-containing) PCs (Herruzo et al. 2019). The reason for this discrepancy is unknown, but may be due to experimental differences in Orc1 depletion, or due to intrinsic differences between yeast strains that were used (Cardoso da Silva et al. 2020; Herruzo et al. 2019). Alternatively, these findings could indicate the existence of more than one (molecularly distinct) pool of Pch2, of which not all depend on Orc1 (see also below).

ORC associates with autonomously replicating sequences (ARSs) present throughout the genome (Bell and Labib 2016). Based on the relationship between Pch2 and ORC, we systematically mapped Pch2 chromosome association during meiotic G2/Prophase. However, despite the confirmed binding of ORC to ARSs during meiotic G2/prophase (Cardoso da Silva et al. 2020; Villar-Fernandez et al. 2020), Pch2 could not be detected at ARS regions (Cardoso da Silva et al. 2020). This could indicate that the interaction between Pch2 and Orc1/ORC does not occur at origins of replication. Interestingly, our analysis revealed an association of Pch2 with the bodies of a subset of RNA Polymerase II-transcribed (RNAPII) genes, distributed along budding yeast chromosomes (Cardoso da Silva et al. 2020). The genes where Pch2 was found to be associated tended to be actively transcribed, but additional molecular determinants that drive association of Pch2 with defined genes remain to be discovered. Recruitment of Pch2 to identified RNAPII genes was impaired in *zip1Δ* cells (Cardoso da Silva et al. 2020), indicating a connection between Pch2, RNAPII sites, and the presence of Zip1 on chromosomes. Crucially, association of Pch2 to actively transcribed genes (assessed via Chromatin Immunoprecipitation (ChIP)) and to synapsed chromosomes (assessed via immunofluorescence of chromosome spreads) is substantially reduced upon acute RNAPII transcription depletion (via nuclear depletion of Rpo21 (Cardoso da Silva et al. 2020), the largest subunit of RNAPII (Geisberg et al. 2014; Haruki et al. 2008; Woychik and Young 1990)). Under these conditions, the recruitment of Pch2 to the rDNA/nucleolus appeared unaffected (Cardoso da Silva et al. 2020), pointing to a specific role for RNAPII in mediating recruitment to euchromatic regions. Is ORC involved in the recruitment of Pch2 to these RNAPII-dependent transcription sites? In consonance with Pch2-binding patterns, association of ORC to coding regions of RNAPII-transcribed genes has been observed in vegetative cells (Shor et al. 2009). Indeed, we detected association of Orc1 (and Orc2, another subunit of ORC) with euchromatic transcribed genes which are bound by Pch2 during meiotic G2/prophase (Cardoso da Silva et al. 2020). Importantly, Pch2 levels associated with defined RNAPII genes were reduced in *orc1-161* cells (Cardoso da Silva et al. 2020). Thus, these data suggest that ORC (and especially Orc1) and RNAPII play a role in the recruitment of Pch2 to meiotic chromosomes in budding yeast (Fig. 1b, c). Our biochemical analysis of the interaction between ORC and Pch2 also revealed that Orc1 is a key component mediating this interaction (Villar-Fernandez et al. 2020). Based on the known biochemical properties of AAA + ATPase interactions with adaptors and clients (Hanson and Whiteheart 2005; Puchades et al. 2020), these collective data suggest that Orc1 might fulfill an adaptor-like role in guiding Pch2 to defined genomic regions during meiotic G2/prophase (Fig. 1b). What is the connection between Orc1 and the functional effect of Pch2 on its substrate Hop1? Impairing Orc1 triggers Pch2-like phenotypes (such as increased DSB activity) within the rDNA (San-Segundo and Roeder 1999; Vader et al. 2011) which have been associated with a failure to remove Hop1 (San-Segundo and Roeder 1999). However, within euchromatin, interfering with Orc1 function [which in one study was shown to lead to a reduction of Pch2 recruitment to euchromatin (Cardoso da Silva et al. 2020)] did not lead to effects on Hop1 chromosomal abundance (Cardoso da Silva et al. 2020; Herruzo et al. 2019). These observations imply that within euchromatin, (i) additional factors influence Pch2 function, and/or (ii) more than one Pch2 chromosomal pool (potentially one that interacts with Xrs2 (Ho and Burgess 2011)) exists, which can control the localization of Hop1.

## Pch2 recruitment: a connection to chromatin modifications?

Now that we have a better understanding of the regions within the genome where Pch2 is recruited, can we generate a comprehensive model describing the factors that regulate Pch2—including their interplay? Several lines of evidence point to a relationship between chromatin modifications (potentially mediated by active transcription), and Pch2 (see above). As such, Pch2 recruitment might be influenced by a specific (transcription-associated) histone ‘code’, potentially driven by Sir2- and Dot1-dependent activities (Cavero et al. 2016; Ontoso et al. 2013; San-Segundo and Roeder 1999,2000). However, Pch2 does not encode any obvious domain that could function as a chromatin reader, so how could Pch2 be connected to chromatin state? Interestingly, Orc1 contains a Bromo-adjacent homology (BAH) domain—a nucleosome interacting module (Callebaut et al. 1999). The BAH domain of Orc1 contributes to Pch2 function in the nucleolus (Vader et al. 2011), and interactions of Orc1–BAH with the nucleosome core particle are essential for this function (De Ioannes et al. 2019). Removal of the BAH domain in Orc1 also impairs the binding of Pch2 to regions of transcriptional activity (Cardoso da Silva et al. 2020), implying that Orc1 makes use of its nucleosome reading domain (BAH) to recruit Pch2 to these regions. We note that in vitro analysis of the interaction between Pch2 and Orc1/ORC showed that the BAH domain of Orc1 is not directly involved in establishing the interaction between Pch2 and ORC (Villar-Fernández and Vader, unpublished observations). These findings suggest that a role for the BAH domain of Orc1 in regulating the recruitment of Pch2 is related to its ability to interact with (modified) nucleosomes.

The BAH domain of Orc1 is structurally and evolutionarily related to the BAH domain of Sir3, a component of the yeast SIR complex (Armache et al. 2011; Bell et al. 1995; De Ioannes et al. 2019; Hanner and Rusche 2017; Hickman and Rusche 2010; Kellis et al. 2004). The biochemical characteristic of this BAH domain provides potential clues to the connection between Orc1, transcription, and Pch2 recruitment. Sir3-BAH displays a preference for nucleosomes containing non-acetylated H4K16 histones and interacts with H3K79, in a manner that is negatively influenced by methylation (Armache et al. 2011; Hecht et al. 1995; Liou et al. 2005; Norris and Boeke 2010). These residues in H4 and H3 are substrates of Sir2 and Dot1, respectively—two factors that intriguingly also influence Pch2 recruitment and function (see above) (Fig. 1b, c)(Cavero et al. 2016; Ontoso et al. 2013; San-Segundo and Roeder 1999, 2000). Within silent chromatin, nucleosomes are thought to lack acetylation and methylation, due to a local enrichment of Sir2 and eviction of Dot1 (Cavero et al. 2016; Ng et al. 2003; Norris and Boeke 2010; Srivastava et al. 2016; van Leeuwen et al. 2002; Xue et al. 2015). RNAPII activity is accompanied by defined co-transcriptional histone modifications, and, interestingly, Dot1 activity (and thus the associated methylation of H3K79) is associated with active RNAPII-dependent transcription (Kim et al. 2012; Nguyen and Zhang 2011; Wood et al. 2018). Pch2-binding sites (associated with RNAPII activity) correlate positively with mono-methylation, but not with di- and trimethylation of H3K79 (Cardoso da Silva et al. 2020). It can thus be speculated that Pch2-binding genes display certain H3K79/H4K16 histone profiles that are ‘read’ by Orc1-BAH domain, potentially providing a rationale for the association of Pch2 to a selected group of RNAPII-transcribed genes, and the involvement of Orc1 (and its BAH domain) (Cardoso da Silva et al. 2020). In cells that lack Dot1 activity (and thus methylation of H3K79), Pch2 localization is restored in cells that lack Zip1 (Ontoso et al. 2013; San-Segundo and Roeder 2000), suggesting that Dot1 might negatively regulate Pch2 recruitment. In the absence of Zip1, removal of Sir2 similarly allows the association of Pch2 with meiotic chromosomes (San-Segundo and Roeder 1999). We note that Sir2 is present and active at euchromatic origins of replication (Hoggard et al. 2020, 2018), suggesting that it might play yet unappreciated roles within euchromatin during meiosis. Clearly, revealing the binding patterns of Pch2 under conditions where Dot1/Sir2 activity is impaired might provide insights into the connection between Pch2 and Dot1/Sir2 activity. Together, these observations warrant careful comparisons of different H3K79 methylation/H4K16 acetylation profiles in relation to Pch2-binding sites during meiotic G2/prophase, to reveal epigenetic profiles that might influence Pch2/Orc1 association. Genome-wide analyses should be coupled to in vitro biochemical analysis of the interactions between Pch2, Orc1 (and its BAH domain), and (modified) nucleosomes. The recent reconstitution of the Pch2/ORC interaction (Villar-Fernandez et al. 2020), combined with the ability to generate specifically modified nucleosomes (Simon et al. 2007), should allow such analyses. In conclusion, these data collectively indicate that a key determinant of Pch2 recruitment to euchromatin is likely provided by defined histone modifications. The BAH domain of Orc1, as a ‘reader’ of such modifications, might represent a key mediator that enables Pch2 recruitment. Under such a model, the recruitment of Pch2 to the silent chromatin of the rDNA as well as euchromatin would be founded on a shared molecular basis, where key contributions are made by Orc1/ORC and defined chromatin modifications (Fig. 1b, c).

## Role of chromosome organization and topology in Pch2 recruitment

Despite the recent data emphasizing an intimate molecular relationship between (specific and local) chromatin modifications and Pch2, other lines of evidence also point to roles for chromosome organization influencing Pch2 recruitment. Most prominently is the role played by the SC component Zip1: without this factor, Pch2 is not recruited to euchromatin. However, recent work has begun to reveal further connections between Pch2 localization and chromosome organization, metabolism, and architecture. Important chromosomal processes such as replication fork movement, RNAPII transcriptional elongation, and recombination can lead to topological stress which can be alleviated by DNA topoisomerases (Baranello et al. 2016). A recent report found that interfering with topoisomerase II (Top*2*) function impairs efficient recruitment of Pch2 to chromosomes (Heldrich et al. 2020). An earlier study also reported a role for the nucleoporin Nup2 in promoting the localization of Pch2 (Subramanian et al. 2019) (Fig. 1b, c), although it remains unclear how direct the connection between Pch2 and Nup2 is. As a component of the Nuclear Pore Complex (NPC), Nup2 is implicated in chromosome organization by establishing chromatin boundaries, while also having a role in NPC-mediated regulation of RNAPII-dependent transcription (Brickner et al. 2019; Casolari et al. 2004; Dilworth et al. 2005; Ptak and Wozniak 2014, 2016). In cycling cells, tethering of RNAPII genes at the nuclear periphery (by Nup2) depends on gene activity (Brickner et al. 2012; Schmid et al. 2006). It is unknown if actively transcribed genes are tethered to nuclear pores during meiotic G2/prophase by Nup2, and it will be important to assess if Pch2 (and Pch2-associated chromosomal regions) are found in close proximity to nuclear pores. In *D. melanogaster*, Pch2 localizes adjacent to the nuclear envelope (Joyce and McKim 2010), suggesting a potential connection between the nuclear envelope and Pch2 function during meiosis in this organism. A specific role for NPCs might explain the effect of Nup2 deletion on Pch2 recruitment (Subramanian et al. 2019). If so, RNAPII depletion might affect Pch2 recruitment through an effect on the tethering of transcribed genes to nuclear pores. Alternatively, as suggested by Subramanian and co-workers (Subramanian et al. 2019), Nup2 could influence Pch2 recruitment via an effect on Sir2 function (potentially within the nucleolus). Such a model would suggest that crosstalk between chromosome architecture and chromatin modification in influencing Pch2 recruitment. Impairing Top2 function might create topological constraints that similarly affect the tethering of high-transcribed genes to nuclear pores (Bermejo et al. 2011), thus impairing Pch2 localization. As such, it is possible that effects that are observed on Pch2 in mutants that affect different aspects of chromosome architecture (i.e., NPC-tethering, topological stress, and active transcription) converge on a shared molecular basis (Cardoso da Silva et al. 2020; Heldrich et al. 2020; Subramanian et al. 2019) (Fig. 1c).

During meiotic G2/prophase, chromosomes are organized into a specialized loop-axis arrangement (driven by chromosome axis factors, see above), upon which the SC eventually assembles. All other chromosomal processes (i.e., transcriptional activity, DSB formation, and recombination) are executed in the context of this highly specialized architecture. Previous studies have highlighted the role of RNAPII-driven transcription in axis positioning, and it has been proposed that RNAPII genes are localized within chromatin loops (of meiotic chromosomes) (Bonev and Cavalli 2016; Muller et al. 2018; Schalbetter et al. 2019; Sun et al. 2015). The observed occupancy of Pch2 to RNAPII-associated genes thus suggests that a pool of Pch2 may reside within loops that emanate from the chromosome axis (Fig. 1c). Taken as a whole, it is tempting to speculate about the existence of a pathway in which several players involved in diverse aspects of chromosome organization converge on the regulation of Pch2 recruitment, especially in relation to active RNAPII-dependent transcription. This particular localization pattern might also have implications for the connection between this pool of Pch2 and its substrate Hop1. Hop1 is located at the chromosome axis (Panizza et al. 2011), and a comparison of Pch2-binding sites with axis-associated proteins (Rec8, Red1, and Hop1) revealed a distinct binding profile with little overlap between Pch2 and axis sites (Cardoso da Silva et al. 2020). This suggests that the RNAPII-associated pool of Pch2 might therefore not be able to remove axial Hop1, and implies the existence of another pool of Pch2 which could be involved in the removal of Hop1 from axial sites.

Many of the molecular processes that influence Pch2 recruitment in meiotic G2/prophase are also active in vegetative cells. However, we found that ectopic expression of Pch2 in vegetatively growing cells—Pch2 expression is normally restricted to meiosis in budding yeast—is not sufficient to trigger the recruitment of Pch2 to its mapped binding sites (Cardoso da Silva et al. 2020), highlighting the important role played by meiosis-specific events (i.e., Zip1/SC assembly). Under *NUP2-, TOP2-,* and RNAP-II (*RPO21*)-inhibited conditions in meiotic G2/prophase, the effect on Pch2 recruitment was not accompanied by observable defects on the establishment of chromosome synapsis (Cardoso da Silva et al. 2020; Heldrich et al. 2020; Subramanian et al. 2019). These findings suggest that a meiosis-specific event (likely centered around SC formation) acts in combination with general events controlling chromosome metabolism to generate a chromatin environment that is permissive for binding of Pch2 to euchromatin. A tantalizing idea is that Pch2 (potentially in collaboration with Orc1) is responsive to a certain type of chromosome architecture or stress, as such only being recruited to (regions of) meiotic chromosomes that experience such a state. A recruitment mode like this would not be without precedent: for example, the PICH helicase is recruited to so-called ultra-fine DNA bridges (UFBs) in anaphase, in a manner that is responsive to the amount of mechanical stress present on such UFBs (Biebricher et al. 2013; Liu et al. 2014; Sarlos et al. 2018). Interestingly, the accumulation and (re)distribution of mechanical stress on meiotic chromosomes has been put forward as a factor that influences chromosome metabolism in meiotic G2/prophase, and, as an example, Top2 plays a role in this process (Zhang et al. 2014a, b). Finally, we note that it remains possible that the role played by chromosome architecture and topology with respect to Pch2 recruitment might be related to the observed connection between Pch2 and chromatin state (Subramanian et al. 2019). Clearly, a major focus should be on elucidating the interplay between chromosome architecture, the specific role of SC establishment, chromatin modifications, and Pch2 recruitment.

## Future directions

Despite the recent progress in our understanding of the mechanisms and factors behind Pch2 recruitment, many questions remain. Clearly, many pieces of evidence point to a seemingly complex relationship between chromosome structure, the SC, and Pch2 recruitment. It will be crucial to reveal the role played by the SC: are other factors (like Ecm11 and Gmc2) also involved in Pch2 recruitment? How does Zip1 enable the recruitment of Pch2 and what is the connection between SICs, SC establishment, chromosome structure and Pch2 recruitment? Is its role connected to the meiosis-specific re-organization of chromosomes (in connection to loop-axis establishment, topology, and transcriptional activity), or does it involve a more ‘direct’ role in Pch2 recruitment, potentially via SC-associated post-translation (chromatin) modifications or even through defined molecular interactions? What is the exact defect in SC function caused by the *zip1-4LA* mutant (Mitra and Roeder 2007; Subramanian et al. 2016), and how is this molecularly related to Pch2 recruitment?

Certain aspects that influence Pch2 localization (e.g., reliance on Orc1 and influence of histone modifications) are shared between the local recruitment to the rDNA and to euchromatin. As such, and in light of the fact that recruitment of Pch2 to rDNA occurs independently of Zip1/SC assembly, understanding the rules of this recruitment might shine important light onto the association of Pch2 to chromosomal regions where Zip1/SC assembly does play a role (Fig. 1b, c). It will be interesting to investigate, for example, whether active transcription within the rDNA (potentially by the rDNA-associated polymerases RNAPI and RNAPIII) is equally involved in Pch2 recruitment within this specialized chromatin environment. Related to this is the intriguing connection between Pch2, Orc1, and chromatin modifications. Can we understand how chromatin modifications (especially those affected by Sir2 and Dot1 activities) affect Pch2 recruitment? We suggest the use of in vitro biochemistry to understand the interactions between these factors (Pch2 and Orc1/ORC) and nucleosomes as an important avenue that has the potential to provide molecular insights into this intriguing connection. Furthermore, it should be determined whether the contribution of Orc1/ORC to Pch2 recruitment and function is conserved outside of budding yeast (Cardoso da Silva et al. 2020; Vader et al. 2011; Villar-Fernandez et al. 2020).

In addition to these questions, it will be important to reveal more detail on two other aspects of Pch2 recruitment and function. What is the relation between the different modes of recruitment of Pch2 and its described downstream effect on the removal of HORMA-domain-containing proteins? For example, the recruitment of Pch2 to regions of active transcription is not associated with general effects on Hop1 chromosomal function (Cardoso da Silva et al. 2020). Does this suggest that this pool of Pch2 might play an as of yet unknown role and that more than one (molecularly unique) pool of Pch2 might exist (Fig. 1c)? It is also interesting to note that recent work has found that Hop1 removal along chromosomes is not a uniform process, with certain chromosomal regions appearing refractory to removal of Hop1 (Subramanian et al. 2019). This could suggest the existence of distinct (regional) pools of Pch2 which might differentially affect the chromosomal association of Hop1. In light of this, the described (functional) interaction between Pch2 and Xrs2 (Ho and Burgess 2011) should be explored in more detail (Fig. 1c). Clearly, we need to delve deeper into these questions in order to understand the molecular mechanisms that drive Pch2 recruitment. Finally, although several aspects of the Pch2 chromosomal recruitment and function seem conserved (such as a clear dependence on Zip1 (and its homologs) and an effect on chromosomal HORMA proteins), to what level are additional pathways shared, or where does the regulation diverge between species?

As a whole, revealing the fascinating and seemingly complex regulation of Pch2 recruitment to chromosomes will be important to deepen our understanding between this key meiotic regulator and the processes that enable DNA break formation and recombination during meiotic G2/prophase.
